# Scaling-up the Systems Analysis and Improvement Approach for prevention of mother-to-child HIV transmission in Mozambique (SAIA-SCALE): a stepped-wedge cluster randomized trial

**DOI:** 10.1186/s13012-019-0889-z

**Published:** 2019-04-27

**Authors:** Kenneth Sherr, Kristjana Ásbjörnsdóttir, Jonny Crocker, Joana Coutinho, Maria de Fatima Cuembelo, Esperança Tavede, Nélia Manaca, Keshet Ronen, Felipe Murgorgo, Ruanne Barnabas, Grace John-Stewart, Sarah Holte, Bryan J. Weiner, James Pfeiffer, Sarah Gimbel

**Affiliations:** 1 Department of Global Health, University of Washington Schools of Medicine and Public Health, 1705 NE Pacific St, Seattle, WA 98195 USA; 2 Health Alliance International (HAI), 1107 NE 45th St, Suite 350, Seattle, WA 98105 USA; 3 Department of Epidemiology, University of Washington School of Public Health, 1705 NE Pacific St, Seattle, WA 98195 USA; 4 Community Health Department, School of Medicine, Eduardo Mondlane University, Avenida Salvador Allende, 702 Maputo, Mozambique; 5 Manica Provincial Health Department, Ave 25 de Setembro, Chimoio, Mozambique; 6 Fred Hutchinson Cancer Research Center, 1100 Fairview Ave N, Seattle, WA 98109 USA; 7 Department of Family and Child Nursing, University of Washington School of Nursing, 1959 NE Pacific St, Seattle, WA 98195 USA

**Keywords:** Systems analysis and improvement approach (SAIA), PMTCT, RE-AIM, CFIR, ORIC, Process mapping, Cascade analysis, Continuous quality improvement, Stepped wedge, Implementation science

## Abstract

**Background:**

The introduction of option B+—rapid initiation of lifelong antiretroviral therapy regardless of disease status for HIV-infected pregnant and breastfeeding women—can dramatically reduce HIV transmission during pregnancy, birth, and breastfeeding. Despite significant investments to scale-up Option B+, results have been mixed, with high rates of loss to follow-up, sub-optimal viral suppression, continued pediatric HIV transmission, and HIV-associated maternal morbidity. The Systems Analysis and Improvement Approach (SAIA) cluster randomized trial demonstrated that a package of systems engineering tools improved flow through the prevention of mother-to-child HIV transmission (PMTCT) cascade. This five-step, facility-level intervention is designed to improve understanding of gaps (cascade analysis), guide identification and prioritization of low-cost workflow modifications (process mapping), and iteratively test and redesign these modifications (continuous quality improvement). This protocol describes a novel model for SAIA delivery (SAIA-SCALE) led by district nurse supervisors (rather than research nurses), and evaluation procedures, to serve as a foundation for national scale-up.

**Methods:**

The SAIA-SCALE stepped wedge trial includes three implementation waves, each 12 months in duration. Districts are the unit of assignment, with four districts randomly assigned per wave, covering all 12 districts in Manica province, Mozambique. In each district, the three highest volume health facilities will receive the SAIA-SCALE intervention (totaling 36 intervention facilities). The RE-AIM framework will guide SAIA-SCALE’s evaluation. Reach describes the proportion of clinics and population in Manica province reached, and sub-groups not reached. Effectiveness assesses impact on PMTCT process measures and patient-level outcomes. Adoption describes the proportion of districts/clinics adopting SAIA-SCALE, and determinants of adoption using the Organizational Readiness for Implementing Change (ORIC) tool. Implementation will identify SAIA-SCALE core elements and determinants of successful implementation using the Consolidated Framework for Implementation Research (CFIR). Maintenance describes the proportion of districts sustaining the intervention. We will also estimate the budget and program impact from the payer perspective for national scale-up.

**Discussion:**

SAIA packages user-friendly systems engineering tools to guide decision-making by frontline health workers, and to identify low-cost, contextually appropriate PMTCT improvement strategies. By integrating SAIA delivery into routine management structures, this pragmatic trial is designed to test a model for national intervention scale-up.

**Trial registration:**

ClinicalTrials.gov NCT03425136 (registered 02/06/2018).

**Electronic supplementary material:**

The online version of this article (10.1186/s13012-019-0889-z) contains supplementary material, which is available to authorized users.

## Background

UNAIDS goals to have 90% of HIV-infected individuals diagnosed, 90% linked to care and on treatment, and 90% virally suppressed are largely not being met for pregnant women. In Mozambique—a resource-limited setting with high HIV prevalence—94% of HIV-infected pregnant women are diagnosed and 92% initiate treatment [1], but over 40% do not return for follow-up visits after initiating antiretroviral therapy (ART) in pregnancy [2]. The combination of undiagnosed, untreated, and poorly controlled HIV infection results in high mother-to-child HIV transmission, and high maternal morbidity.

Recognizing the potential benefits of early ART initiation in pregnancy, the World Health Organization (WHO) amended its guidelines in 2013 to recommend that countries with generalized HIV epidemics adopt Option B+ (lifelong ART to all HIV-infected pregnant and postpartum women regardless of CD4 count–Option B+) [3]. Sub-Saharan African countries rapidly implemented Option B+, and access to ART increased substantially. In Mozambique, the proportion of pregnant women in PMTCT initiating ART increased from 10 to 50% in the year of Option B+ introduction, and achieved over 90% in 2015 [1].

Though PMTCT services have achieved scale, quality gaps constrain adherence and viral suppression, leaving children at risk for acquiring HIV [4]. In central Mozambique, 58% of women in Option B+ are lost to follow-up by 6 weeks postpartum, and only 37% remain in care by 6 months postpartum [5]. Of those retained in care, less than half are virally suppressed (< 1000 copies/mL) at their first postpartum visit.

Health system weaknesses are important barriers to Option B+ effectiveness [6]. Factors such as long wait and short consult times [7], unreliable medicine and supply systems [8], and poor counseling impede support to patients to remain in care and adhere to ART [9]. Improving PMTCT service efficiency may address performance gaps by providing more time for limited human resources to interact with patients, improving integration across services, and reduce missed opportunities that lead to loss to follow-up, poor ART adherence, mother-to-child HIV transmission, and poor maternal health outcomes.

The priority for improving the effectiveness of PMTCT services is to increase the number of women-infant pairs successfully passing through the multiple, sequential steps in the PMTCT cascade [10], and ensuring their access to efficacious interventions. Structural implementation strategies—like the Systems Analysis and Improvement Approach (SAIA)—improve PMTCT effectiveness, and are potentially scalable across public sector health systems. Such novel systems engineering approaches have been increasingly applied in health care to improve program effectiveness by identifying and reducing system inefficiencies in complex, multi-step health services, such as the PMTCT cascade. These tools, including process mapping and continuous quality improvement, have been adapted from systems engineering manufacturing improvement, and have led to increases in efficiency through simple, low cost, iterative adaptations in service delivery design [11]. Systems engineering encourages participation of frontline staff to analyze systems, and to define, implement, and evaluate improvement strategies; and with involvement of senior management champions, has been shown to lead to appropriate, effective, and sustainable solutions, improved health service delivery, and improved patient outcomes [12–14].

Experience with systems engineering is growing in resource-constrained settings [15, 16], including in applications to PMTCT [17–19]. In a previous cluster randomized trial, we found our systems engineering strategy (SAIA) [20] improved PMTCT performance [21] and was highly accessible and user-friendly for frontline health workers and managers [22]. The SAIA implementation strategy enables frontline staff to gain a comprehensive view of their complex delivery systems, identify target areas to improve, and iteratively test modifications to improve the quality and quantity of system outputs and patient health outcomes. Participants noted that SAIA was practical and easy to use; stimulated communication, consensus decision-making, and accountability across multiple service points in their facility (e.g., antenatal, maternity, postpartum, laboratory, and pharmacy); and was accessible by relying on routine data to guide decision-making in real-world service delivery environments [22]. SAIA is flexible, and can be applied through routine management systems to lead to enduring improvements in cascade effectiveness.

Despite evidence on the effectiveness of systems engineering, research on strategies to scale-up these improvement strategies is lacking. Systems engineering approaches must be expanded using models that are scalable (achieve broad scale across delivery systems), affordable (require modest resources to align with current health sector investments), sustainable (integrated into and led by public sector health systems), and effective (retain active ingredients for impact). Without research on how to scale-up evidence-based structural implementation strategies for PMTCT through routine management systems, we will fail to address poor performance and to maximize significant investments in Option B+.

### Goals and objectives

The overall goal of this study is to test the effectiveness of a dissemination and implementation model for the SAIA intervention (SAIA-SCALE) that is delivered by district maternal and child health supervisors (rather than research nurses) to serve as a foundation for national scale-up. We selected districts as the unit of intervention allocation as district management structures link health systems (which allocate human resources, medical supplies and financial resources, and provide oversight and support to ensure strong governance and foster accountability) and health facilities (where services are delivered). Districts are the logical disseminating vehicle for structural interventions as they have oversight responsibility for services within their districts, ability to access resources to meet health facility needs, and authority to put in place management decisions that affect the environment within subordinate health facilities. By focusing on testing a novel approach to scale the SAIA implementation strategy through routine management structures, the study is designed to address critical gaps in the field of implementation science. SAIA-SCALE’s specific objectives are to:

1. Develop a district-based dissemination and implementation strategy for SAIA (SAIA-SCALE), using the RE-AIM model to evaluate the program’s Reach, Effectiveness, Adoption, Implementation, and Maintenance; and
2. Estimate the budget and program impact from the payer perspective to scale-up SAIA compared to the standard of care using activity based micro-costing and mathematical models of HIV transmission.

**Fig. 1:**
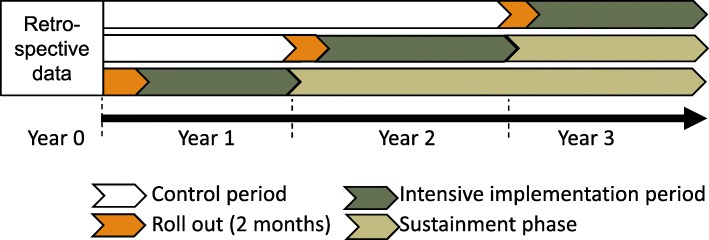
SAIA-SCALE stepped wedge implementation timeline

SAIA-SCALE employs a stepped wedge cluster randomized trial implementation schedule to randomly allocate all 12 districts in Manica province into three implementation waves, staggered by 1 year (3 years total). District maternal and child health supervisors deliver the intervention in three health facilities per district (36 total) during a 1-year intensive phase (mentored by research nurses experienced with the intervention from the SAIA trial), with subsequent implementation led independently by the district supervisors (sustainment phase) (Fig. 1). Our design has a number of advantages for this study. First, a phased-in design will ensure that all 12 districts in Manica province will receive the evidence-based SAIA implementation strategy. Second, the design will assess SAIA’s performance under pragmatic conditions. Third, a randomized design reduces potential confounding, and provides robust evidence of SAIA-SCALE’s impact. Fourth, the design is appropriate to assess the dissemination strategy for SAIA-SCALE (delivery by district health system managers), while assessing impact on the facility and patient levels. Finally, the design allows for assessing maturation patterns in SAIA-SCALE’s effects over time, including during the intensive and sustainment phases.

## Methods

### Description of the SAIA implementation strategy

The core elements of the SAIA implementation strategy have been previously described [20]. The strategy includes a package of systems engineering and improvement tools that are delivered iteratively at the health facility level by trained mentors. SAIA provides facility-level PMTCT staff and managers with a holistic view of their systems’ performance, highlights gaps, prioritizes cascade steps for improvement, identifies modifiable processes for improvement, and tests contextually appropriate solutions. The approach is designed to be user friendly, relies on routinely reported data, and engages clinic teams in consensus decision-making to lead to sustainable solutions that are within their control and adapted to the clinic context.

**Fig. 2:**
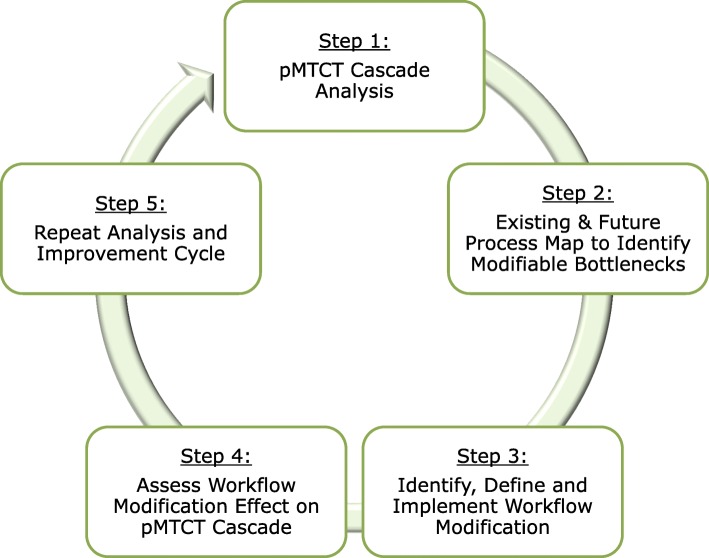
Five steps of the Systems Analysis and Improvement Approach (SAIA) implementation strategy

SAIA is designed as a cyclical, five-step process (Fig. 2):

- Step 1: Understand PMTCT performance and identify priority areas for improvement. The PMTCT cascade analysis tool (PCAT) [23] (Additional file 1: Figure S1) uses routine data to provide a rapid, systems-level view of drop-offs along the PMTCT cascade. As an analytic tool, the PCAT provides frontline staff and facility managers with a view of the greatest potential for flow improvements through maternal (antenatal care➔postpartum care) and pediatric (birth➔ART initiation) cascades. As a departure from the original SAIA trial, SAIA-SCALE employs a mobile application of the PCAT that describes the PMTCT cascade using Android-based tablets or cellular phones [24].
- Step 2: Identify facility-level modifiable bottlenecks using process mapping. Enabling facility-level staff to identify and gain consensus on bottlenecks to address in their PMTCT system is essential to defining innovations to implement. SAIA applies sequential process flow mapping [25] (Additional file 2: Figure S2), coupled with workflow observation, to identify bottlenecks and guide discussion on opportunities to modify workflow across PMTCT services.
- Step 3: Define and implement facility-specific workflow adaptations to address modifiable bottlenecks. After identifying modifiable barriers within cascade steps, facility staff identify a simple, specific change to improve performance within the targeted step. Selected workflow adaptations should be feasible to implement, be within the scope of influence of facility management and PMTCT staff, and be expected to lead to rapid, substantial improvements in the targeted cascade step (see Table 1 for examples from the original SAIA trial). Ideas for adaptations come from brainstorming solutions with facility-level staff as well as from modifiable best practices from the SAIA trial, high performing PMTCT services in Mozambique, and the published literature. An implementation plan for the innovation is described in writing to clarify operational design and roles, and ensure clinic-level consensus.
- Step 4: Monitor changes in routine performance and initiate additional iterations. Facility staff monitor improvements in routine data for the PMTCT step selected for improvement. Measuring the absolute increase in the proportion of women and infants progressing through targeted steps captures large, rapid improvements accompanying modifications.
- Step 5: Repeat cycle. Systems engineering process improvements are by definition iterative, with ongoing testing of innovations responsive to evolving, contextually specific barriers. Facility staff repeat steps 1–5, focusing on identifying new approaches to modify previously identified barriers, or if the first cycle was successful, improving priority bottlenecks identified in a repeated systems analysis.

**Table 1:** Facility-level workflow modifications from the original SAIA trial

Type of change	Specific example	Process improvement
1. Service reorganization	CD4 blood draws changed from daily to weekly	HIV+ mothers with CD4 increased from 26% to 56%
2. Expand patient knowledge	Maternity tours provided to ANC patients	16% increase in institutional births
3. Improve communication across the health care team	ANC nurses pick up lab results instead of waiting	Lab return time reduced from 6 weeks to 10 days
4. Improve data and its use	At shift change, head nurse cross checks pharmacy and maternity registries on infant ARV administration	54% to 88% increase in correct administration
5. Introduce new approaches (norms, treatments, modalities, technologies)	(Re) test women with no test or > 3 months prior HIV test, in family planning	98-fold increase in testing after initial ANC screening (limited impact on identification of HIV+ women)

### SAIA-SCALE trial design

This study employs a 3-year, three-wave stepped wedge cluster randomized trial design to assess the impact of the SAIA-SCALE dissemination strategy (Fig. 1; Consort Checklist in Additional file 3). Each wave includes four randomly selected districts, with three health facilities per district purposively selected to receive SAIA. For each district, the first year of implementation will be *intensive* (where district supervisors are accompanied by research nurses experienced with the SAIA strategy). Subsequent years are defined as the *sustainment* phase, where support from research nurses is withdrawn.

### Process for introducing SAIA

SAIA-SCALE’s delivery schedule, intervention guides, and intervention tools are based on the SAIA trial. Immediately prior to initiating implementation, two maternal and child health supervisors from each district initiating implementation in the activating wave receive a 1-week group training on the SAIA strategy led by research nurses from the original SAIA trial. This training covers an introduction to the SAIA analysis and improvement tools, implementation schedule, and data collection procedures. If district management personnel change, newly appointed managers will receive on-the-job training in study procedures from SAIA-SCALE research nurses.

Subsequently, district maternal and child health supervisors, together with study nurses, introduce SAIA to selected health facilities over a 2-day period (Table 2). Within each district, SAIA is introduced one facility at a time, until all three intervention facilities are covered in each district. For the first 2 months post-introduction, district maternal and child health supervisors visit implementing health facilities every 2 weeks, followed by monthly visits through the 10-month intensive implementation phase and 2-year sustainment phase. Throughout the first year, maternal and child health supervisors are accompanied on facility visits by study nurses. Based on the initial SAIA trial, it is expected that analysis and improvement cycles will be approximately monthly, with an average of 12 cycles per year in each facility.

**Table 2:** SAIA introduction schedule

Activity	Day 1 AM	Day 1 PM	Day 2 AM	Day 2 PM
Intro to SAIA	X			
Process mapping (ANC, maternity, postpartum, at-risk care)		X		
PCAT			X	
Feedback session				X
Implementation start				X

*ANC* antenatal care, *HIV* human immunodeficiency virus, *PCAT* PMTCT cascade analysis tool, *SAIA* Systems Analysis and Improvement Approach

Travel for district managers to conduct mentorship visits is channeled through funding agreements with district health directorates. These agreements also include a flexible facility support fund (approximately $1500 per facility annually to support implementation of workflow modifications).

### Study setting, eligibility criteria, and randomization

#### Study setting

Manica province, located in central Mozambique with an estimated population of 2 million, includes 12 districts and 118 health facilities (Table 3). Utilization of public sector primary care services is high, with over 92% of women attending at least one antenatal care visit in the previous pregnancy, and 70% delivering in a health facility [26]. In central Mozambique, over 98% of utilization of formal health services is estimated to be in the public sector [27].

**Table 3:** Population, health facility network, and utilization patterns by district in Manica province

District	Pop (2017)^a^	MOH clinics	Clinics with Option B+		1st ANC visits^b^	HIV+ in ANC^b^
	*N*	*N*	*N*	(%)	*N*	%
Barue	185,179	14	7	(50)	10,707	6.5%
Chimoio City	372,821	7	7	(100)	23,543	13.8%
Gondola	201,735	8	5	(63)	16,842	9.3%
Guro	96,930	11	7	(64)	5599	6.0%
Macate	85,062	6	3	(50)	5625	8.6%
Machaze	129,099	11	7	(64)	6813	11.9%
Macossa	48,648	5	3	(60)	2159	5.1%
Manica	225,000	17	11	(65)	13,583	11.8%
Mossurize	219,551	11	9	(82)	13,716	7.5%
Sussundenga	168,200	13	7	(54)	11,448	7.8%
Tambara	54,948	7	4	(57)	4399	5.4%
Vanduzi	124,064	8	3	(38)	5821	10.9%
Total	1,911,237	118	73	(62)	120,255	9.2%

^a^Estimates from 2017 census

^b^2015 annual estimates from routine health management information system

Manica has high HIV prevalence in adults (13.5% compared with 13.2% nationally) [26]. Though all facilities offer HIV testing in antenatal care and provision of antiretroviral medicines to prevent mother-to-child HIV transmission, Option B+ has targeted facilities with higher patient volume and larger capacity, and covers over 60% of facilities in the province.

#### Eligibility criteria

**Fig. 3:**
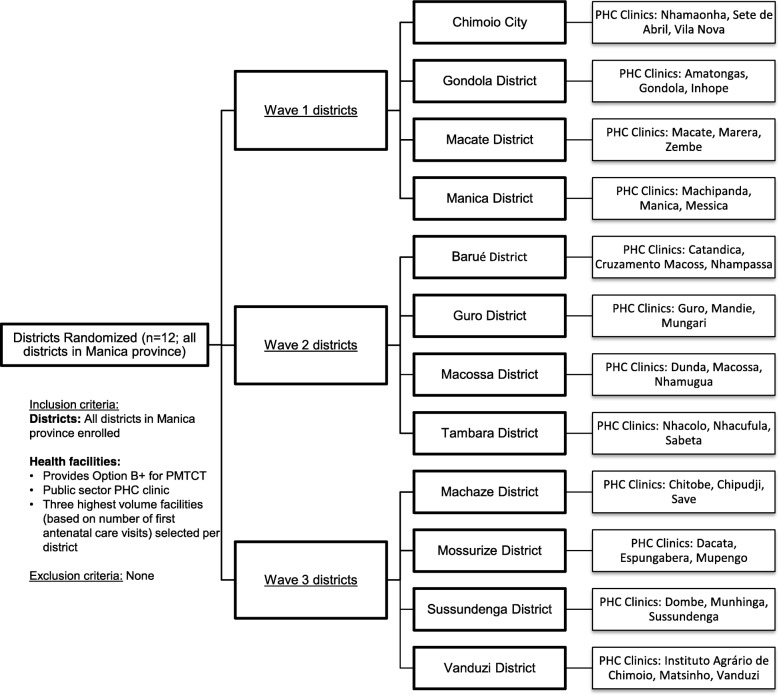
Facility eligibility and randomization. *PHC* primary health care, *PMTCT* prevention of mother-to-child transmission (of HIV)

All 12 districts in Manica province are included in SAIA-SCALE (Fig. 3). In each district, public sector health facilities with Option B+ services were ranked according to patient volume (number of first antenatal care visits in the year prior to randomization), and the highest 3 were purposively selected for study inclusion. This aligns with the Option B+ roll-out to the largest volume facilities with higher burden of HIV.

#### Randomization

Districts were randomized without restriction into implementation waves during a meeting with provincial health authorities and study leadership in Manica province in October, 2017. Districts were randomized prior to selection of intervention facilities. Though already allocated, second and third wave districts have not been informed of the implementation timeframe for their district.

### SAIA-SCALE impact assessment: application of the RE-AIM framework

A mixed-methods evaluation framed by the RE-AIM framework will be used to gain a comprehensive understanding of SAIA-SCALE’s effects. RE-AIM guides assessments of the public health impacts of complex interventions, capturing formative, process, and outcome dimensions on the individual, organizational, and policy levels [28]. Comprehensive assessment of SAIA-SCALE using established implementation science methods gives a multi-dimensional understanding of the active ingredients that define public health impact, and provides actionable information on which to base post-trial expansion planning.

#### Reach

As a descriptive sub-aim, we will use reports from study personnel to estimate the proportion of all public sector health facilities in Manica province reached by SAIA-SCALE (target: 32%, or 36 of the 113 facilities in the province). Using routinely available health management information systems data, the number and proportion of HIV-infected pregnant women and exposed infants reached by the intervention across Manica province will be estimated (the number of woman-infant pairs attending PMTCT services in intervention facilities/total number of estimated eligible woman-infant pairs in the province; target: 80%). This analysis will identify sub-groups of woman-infant pairs whose needs are not met, including (1) those who attend facilities not covered by SAIA-SCALE (estimated using routine health management information system data), or (2) those who do not attend antenatal or maternity services at all (estimated using a mix of availability community survey data and health management information system data).

#### Effectiveness

Intervention effectiveness will assess SAIA-SCALE’s impact on PMTCT cascade (1) facility-level process measures, and (2) patient-level outcome measures.

##### Study population

All Option B+ eligible women accessing care at study facilities, including those diagnosed during pregnancy, delivery, or breastfeeding, as well as those diagnosed prior to presentation at care, are included.

##### Exposure definition

Facilities will be considered unexposed prior to the initiation of the intervention in their districts and exposed thereafter. For the primary analysis, data from the 2-month roll-out phase in each district, during which the intervention will not have achieved full potency, will be excluded (Fig. 1). Individuals’ exposure to the intervention will be based on the exposure status of the facility in the calendar month in which they first entered care and were identified as Option B+ eligible (including those newly initiating ART as well as those already on ART), regardless of whether they presented pre- or postpartum, which reflects that the majority of loss to follow-up occurs between entering care and first ART refill [2].

##### Outcomes

Outcome measures include process measures (to evaluate improved flow across the PMTCT cascade), and individual-level measures of Option B+ effectiveness (Table 4). The primary process outcomes include (1) maternal viral load assessment within 1 month of delivery, and (2) early infant diagnosis by 8 weeks postpartum among HIV-exposed infants, both of which reflect Ministry of Health guidelines in Mozambique. Because of high baseline levels of HIV testing and ART initiation in pregnancy, the former will not be assessed in the trial, while the latter will be considered as a secondary outcome only. Process outcomes are based on monthly aggregate data, and denominators will be the eligible mother-infant pairs presenting at each point in the cascade.

**Table 4:** SAIA-SCALE effectiveness outcome measures

Outcome	Definition (numerator/denominator)	Source
Process measures		
Maternal viral load assessment	#women tested for viral suppression within 1 month of delivery/ #women newly or previously on ART (excluding those lost to follow-up)	ANC MW PPC
Early infant diagnosis (EID) by 8 weeks	#infants tested for HIV by PCR within 8 weeks/ #infants presenting to care within 8 weeks (excluding those lost to follow-up)	CCR
Individual-level measures		
Maternal retention in care at 6 months post ART initiation	Woman retained in care (picked up 6-month pharmacy refill within 15 days of scheduled pickup)/ Enrolled women	ANC PPC
Facility delivery	Woman’s infant delivered in the study facility/ Anticipated deliveries	ANC MW
Maternal ART adherence > 90% (medication possession ratio)	#ART supply days picked up through 3 and 6 months post ART initiation/ #days in 3- and 6-month period	ANC PPC
Viral load suppression at 1 month post delivery	Undetectable viral load (<20 copies/mL)/ Viral load samples drawn within 1 month of delivery	PPC
Mother-to-child HIV transmission rates at 6 months postpartum	Infant tested HIV-positive by 6 months postpartum/ Infants tested for HIV	CCR

Register service location for data abstraction: *ANC* antenatal care, *MW* maternity ward, *PPC* postpartum care, *CCR* at risk child care

The primary Option B+ effectiveness outcome will be maternal retention in care at 6 months post ART initiation. Secondary outcomes will be earlier points along the Option B+ cascade, including facility delivery, maternal adherence to ART as assessed by medication possession ratio (calculated as the total number of day’s supply of ART received divided by the number of days of medicine required) > 90% at 3 and 6 months, viral suppression within 1 month of delivery, and mother-to-child HIV transmission by 6 months postpartum. Individual-level outcomes will only be assessed through 6 months post ART initiation to capture improvements within each step of the trial; however, in the case of mother-to-child HIV transmission, we will also quantify 18-month transmission rates to provide a broader picture of Option B+ effectiveness (these estimates will not be used in assessing SAIA-SCALE impact). Outcome measures are binary, with each woman’s progression through a given step defined as successful or unsuccessful. Progression through earlier steps will not be a prerequisite; women will remain eligible to complete later steps in the cascade despite failing to complete earlier steps.

##### Data sources

Service registries, located at each PMTCT service location (antenatal care, maternity, postpartum care, and at-risk child care for HIV-exposed children), facilitate longitudinal tracking of PMTCT utilization and outcomes for woman-infant pairs. As part of routine care, each woman is assigned a unique identification number that links across service points and clinics, which will be used to abstract registry data for study outcome measures. Retrospective individual-level data for the year prior to the implementation wave will be abstracted in the months following the end of each intervention wave (or in the months prior to wave I to collect baseline data). Ministry of Health administrative data will be used to estimate SAIA-SCALE’s reach across the provincial network of public sector health facilities. Administrative data, together with available community survey data (e.g., the Demographic and Health Surveys) will be used to estimate the number of woman-infant pairs who do not attend antenatal or maternity services.

##### Laboratory measures

Maternal blood samples are drawn at facilities and sent to a central laboratory in the Manica provincial capital for assessment of viral suppression. Results are reported back to facilities in copies/mL and recorded in registries as “detectable/not detectable.” Infant HIV testing is performed using polymerase chain reaction assessment of dried blood spots, analyzed at the provincial laboratory.

##### Power and sample size

We estimate a minimum of 40 Option B+ eligible women will present for a first antenatal care visit each month in each district across the three intervention facilities. For the primary individual-level outcome of 6-month retention in care, with 12 districts in three implementation periods, and assuming an intracluster correlation of 0.2 and α = 0.05, we will have 80% power to detect an increase in retention estimates from 17 to 25%. For the primary facility-level process outcome (infant diagnosis by eight weeks of age among exposed infants), we estimate baseline screening at 42% across the 36 study facilities. Using three, 1-year implementation periods, and targeting 12 districts with three facilities per district, and assuming a mean coverage in control periods of 42%, an intracluster correlation of 0.2 and α = 0.05, we will have 80% power to detect an increase to 78%.

##### Data analysis

Facility-level outcome data will be analyzed using linear mixed-effects models, with clustering by district and an exchangeable correlation matrix. Data for each facility will be aggregated at 2-month intervals for the entire study period, from 1 year pre-intervention through the third year of the intervention. In the primary analysis, facilities will be considered unexposed prior to the introduction of SAIA-Scale and exposed thereafter, and models will be adjusted for study time.

Individual-level outcomes will be analyzed using generalized linear mixed-effects models, with two levels of clustering by facility and district. Individuals’ exposure status will depend on their facilities’ exposure status at the time of their first visit, and models will be adjusted for study time.

For both facility-level and individual-level outcomes, primary analyses will exclude data from the roll-out phase in each district and will consider the intervention effect to be fixed throughout the exposed period. Sensitivity analyses will (a) test for increasing or decreasing effect of the intervention over time and (b) compare the effect of the intervention during the intensive and maintenance phases using interaction terms between exposure status and calendar time or study phase. Effect modification of the intervention effect by facility-level factors such as patient volume, provider training, and distance from the district office will be assessed using interaction terms, with potential effect modifiers assessed categorically or as binary variables depending on distribution. Potential adjustment variables to be assessed include calendar year and individual-level factors such as gestational age at entry into care and timing of HIV diagnosis (during or prior to current pregnancy).

#### Adoption

As an organizational-level measure, we will describe the proportion of intervention districts and facilities that adopt the intervention, defined as attending intervention training and initiating at least one SAIA cycle. Based on the SAIA trial, where 95%of facilities adopted the intervention, we expect high acceptance of SAIA-SCALE (target: 95% adoption).

We will describe determinants of adoption to provide actionable information for further intervention expansion. We will assess organizational readiness for change in intervention clinics within 1 to 3 months after first introducing SAIA in each clinic using the Organizational Readiness for Implementing Change (ORIC) 12-item Likert-type assessment scale [29], and will collect additional information on individuals’ commitment to their facilities, workplace climate, and perceived study buy-in by leadership. In each facility, after the study is introduced, we will apply the ORIC and workplace surveys to eight frontline maternal and child health staff working across the PMTCT cascade and facility leadership (*n* = 288). An additional eight managers from each district were interviewed (*n* = 96) to assess district readiness for change. Analyses will test whether sufficient inter-rater reliability and inter-rater agreement exist to aggregate individual responses to the facility and district levels [30–33]. If tests do not justify aggregation, we will use a measure of intra-facility variability in readiness rather than a facility-level mean in our analysis [31, 33].

We will also assess structural readiness for all intervention districts and facilities annually. At the start of each implementation wave, study personnel will apply a questionnaire adapted from the WHO Service Availability and Readiness Assessment [34] and Demographic and Health Survey Service Provision Assessment [35].

#### Implementation

We will employ two strategies to study the implementation process, focusing on the organizational-levels of district and facility.

##### Consolidated framework for implementation research

Following a similar strategy carried out during the SAIA trial [22], we will use the Consolidated Framework for Implementation Research (CFIR) to guide examination of implementation process, define SAIA-SCALE core elements, and describe determinants of success and failure found across implementing districts and facilities. Discussion guides will be developed using available CFIR tools (http://cfirguide.org/) to gather data about select constructs from each of the five CFIR domains using in-depth interviews and focus group discussions with facility and district staff (qualitative), together with review of implementation meeting minutes (qualitative). We will conduct 18 focus group discussions across the 12 districts and 36 focus clinics. Each focus group discussion will consist of 7–10 participants, sufficient to generate conversation without being so large as to become intimidating [36]. Six focus group discussions will be held with district managers (two focus group discussions at the end of each of the three implementation waves, one each for districts with high and low fidelity to the intervention protocol during each time period). Six focus group discussions will be held with facility managers and six with frontline PMTCT nurses (using the same distribution scheme as district managers). Repeating focus group discussions across the intensive and sustainment phases of the intervention will capture the shared experience of disseminating agents and those receiving the intervention as it adapts to the scaled district model. By purposively holding separate focus group discussions for districts with high and low intervention fidelity, we will uncover salient features of successful implementation. To complement the focus group discussions, 84 semi-structured in-depth interviews will be conducted, two each with frontline health workers from antenatal care and maternity/postpartum care at each of the 36 intervention facilities (*n* = 72), and one maternal and child health supervisor in each district (*n* = 12). Supplemental in-depth interviews are included to collect potentially sensitive information that staff members might be hesitant to share in focus group discussion settings, such as opinions that lower ranking staff feel uncomfortable sharing with their superiors, or sensitive issues related to staffing that higher ranking staff members feel uncomfortable discussing with lower-ranking team members. The in-depth interviews will allow for exploration of the individual experience with disseminating and implementing SAIA-SCALE, and capture intervention adaptations over time. From the SAIA trial, we expect that 84 in-depth interviews and 18 focus group discussions will be sufficient to reach saturation [37].

Focus group discussions and in-depth interviews will be conducted in Portuguese by an experienced facilitator/interviewer accompanied by a note-taker (focus group discussions only), audio-recorded, transcribed, and translated into English by staff fluent in both languages. Two primary coders will independently code transcripts from the in-depth interviews and focus group discussions, and meeting minute summaries, coordinating their coding to create a codebook and conduct thematic analysis.

##### Implementation fidelity to design

We will prospectively document fidelity to protocol with tools from the SAIA trial and tracking implementation using tablet-based surveys filled out by study nurses and district supervisors during facility visits. We will capture the number of planning sessions, participants in these sessions, number of workflow modifications tested, and content and results of these modifications.

#### Maintenance

We will measure the extent to which SAIA-SCALE is institutionalized and sustained over time at the organizational level, focusing on the proportion of districts and facilities continuing to implement the intervention as designed. Our design that includes both “intensive” and “maintenance” phases enables evaluation of how SAIA-SCALE is sustained after the intensive intervention phase. Two approaches will be used to measure maintenance. First, we will describe the proportion of districts and facilities continuing to implement SAIA-SCALE at 12, 24, and 36 months post introduction (target: > 90% at 12 months, > 80% at 24 months, and > 65% at 36 months). Continued implementation is defined as holding monthly SAIA-SCALE meetings. Second, through the in-depth interviews and focus group discussions, we will probe district and facility staff perspectives on determinants of sustained implementation.

### SAIA-SCALE economic evaluation

Using activity-based micro-costing and mathematical models of HIV transmission, we will estimate the budget and program impact from the payer perspective to scale-up the SAIA implementation strategy compared to the standard of care. Mathematical models will simulate health outcomes from study data and literature to estimate clinical outcomes beyond the study province. SAIA-SCALE effectiveness will be estimated from trial results, and varied in sensitivity analyses. Models will account for national guidelines for ART initiation. We will validate the model using Mozambique demographic survey data on HIV incidence and prevalence [26], and estimate the impact of scenarios of national SAIA-SCALE expansion on (1) the proportion of HIV-infected pregnant women initiating ART in pregnancy with suppressed viral load, (2) incident HIV cases in HIV-exposed children, (3) incident infections among partners (secondary transmission), and (4) HIV-related deaths averted. Different expansion scenarios will be based on (1) rate of expansion, (2) breadth of expansion (number/characteristics of future districts), and (3) depth of expansion (number/characteristics of facilities targeted in future expansion). We will estimate incremental costs, and treatment costs incurred and averted, due to the intervention. Costs collected at point of implementation through electronic data capture integrated into district supervisors’ routine activities, and from the study budget, include human resources, training, supplies, intervention delivery, facility support for workflow modifications, etc. Personnel time for SAIA-SCALE tasks will be estimated based on activity tracking and surveys administered to study nurses and district supervisors.

#### Modeling procedures

We will develop a compartmental, deterministic disease progression and transmission model based on previous HIV models using R software [38, 39], reflecting the natural history of mother-to-child HIV infection and disease progression under different ART scenarios [40–43]. The model population will consist of women, woman-infant pairs, and men stratified by retention in care and ART adherence. Population and HIV-specific birth and death rates will be used. As individuals age, they can move to different activity groups at 6-month intervals (the model’s time step). The model captures HIV transmission from infected to susceptible individuals by estimating the *force of infection* [39], or the probability of infection using a heterogeneous pattern of sexual mixing by age and activity class, the proportion of infected individuals in the population, and HIV transmission probability. Each scale-up scenario modeled will assume a change in the proportion of women achieving and sustaining viral suppression, based on the study results and varied in sensitivity analyses. Partial differential equations describing the transition between model states are then developed and solved numerically.

#### Outcomes

Outcomes to be modeled include cost per HIV-infected woman achieving viral suppression. Cost-effectiveness results will report the incremental cost effectiveness ratio (ICER) per incident HIV infection, HIV-related death, and disability adjusted life year (DALY) averted of each scale-up scenario compared to standard care. Interventions will be evaluated based on costs and incremental cost-effectiveness using DALYs to capture the gap between current and ideal health [44]. WHO guidelines will be used to facilitate comparison of cost-effectiveness relative to other strategies [45] Analysis will be from the *payer*/*programmatic perspective*, using data on economic productivity for HIV disability averted and costs of accessing services. Scale-up scenarios will include estimates of the budgetary impact, reporting annual costs disaggregated by budget cost category to inform planning.

### Trial status

SAIA-SCALE was launched in first wave districts in April, 2018, and will be sequentially introduced to wave two districts in April, 2019, and wave three districts in April, 2020.

## Discussion

SAIA-SCALE is a pragmatic trial to test a novel, scalable dissemination approach designed to lead to broad improvements in implementation of Option B+ to reduce mother-to-child HIV transmission across a large health system network. The implementation approach aligns with existing administrative structures, providing a practical tool to integrate into routine functions of district supervisors. As many countries decentralize authority to jurisdictions closer to where services are offered (e.g., districts, sub-districts, and counties), the SAIA-SCALE model is an approach that can enable health authorities to facilitate improvements in their facility network. The phased-in design has a number of advantages. It ensures that all districts receive SAIA; allows for understanding SAIA’s impact during periods of intensive implementation and sustainment of effect when study personnel withdraw their support; and is feasible given the large number of districts and facilities in the study province. The stepped wedge cluster randomized design represents a rigorous approach that is sufficiently powered to detect improvements in process and patient-level outcomes, and reduces sources of bias. The inclusion of multiple implementation science frameworks provides a comprehensive understanding of the implementation process, identifies core intervention components, identifies determinants of successful implementation, and improves transferability of the SAIA-SCALE approach to other settings. The economic evaluation provides practical information for the Ministry of Health in Mozambique to further spread SAIA, and to validate an approach to integrating electronic data capture and activity-based costing into large-scale government programs. Ultimately, the study is uniquely positioned to fill a critical evidence gap on integrating and scaling-up implementation strategies across management systems to optimize PMTCT services across broad facility networks.

The SAIA implementation strategy is user-friendly and low-cost approach to deliver a package of systems engineering tools to improve complex care cascades that require progression through multiple sequential steps, with linkage to other services within and outside of primary care facilities. The strategy has proven to be flexible to content and context, and we are currently adapting SAIA for a number of complex cascades beyond the PMTCT in primary care settings in east and southern Africa (including the pediatric HIV testing and linked care cascade, the services for severe mental illness cascade, the hypertension screening and management cascade, the HIV testing in family planning services cascade, and the cervical cancer screening and treatment cascade). SAIA-SCALE represents the first attempt to study an approach to deliver SAIA, which is a precursor to enabling further spread.

At the time of submission, SAIA-SCALE is in month 12 of implementation in the first four intervention districts and 12 intervention facilities. There has been tremendous enthusiasm for the study among provincial, district, and health facility leadership and staff. Given that district maternal and child health supervisors are delivering the intervention to facilities in their districts, buy-in from authorities is essential for an effective approach and further scaling-up.

Contributions to the literature • Research on implementation strategies generally focus on whether or not they improve adoption and/or implementation of evidence-based interventions. Relatively little research has focused on how to scale-up effective implementation strategies through routine management structures.

• Research on systems engineering tools has largely come from high-resourced health systems, with little from low-income countries.

• Our study examines if delivery of a user-friendly, low-cost package of systems engineering tools through district health teams in a low-income public sector health system improves health system performance and patient outcomes, and provide decision-makers with evidence they need to decide whether and how to further scale-up the approach.

## Additional files

Additional file 1: **Figure S1.** PMTCT Cascade Analysis Tool (PCAT). Legend: Demonstrates number lost and potential gains per step (if that step improved to 100%, holding the other steps constant) for the ANC➔maternity (yellow), and postpartum (blue) cascades. ANC: Antental care; cART: Combination antiretroviral therapy; dx: Diagnosed; PPO: Prophylaxis; (PPTX 45 kb)

Additional file 2: **Figure S2.** Example of PMTCT process maps from two facilities in central Mozambique. Legend: Maps are from a medium-sized rural health center (Tica) and large urban health center (Munhava) in 2009, and demonstrate the flow of women from entry into antental care through receipt of antiretroviral prophylaxis or combination antiretroviral therapy. (PPTX 129 kb)

Additional file 3: CONSORT 2010 checklist for cluster randomized trials. (DOCX 35 kb)

## Abbreviations

ART: Antiretroviral therapy
CD4: Cluster of differentiation 4 (laboratory test)
CFIR: Consolidated Framework for Implementation Research
DALY: Disability adjusted life year
HIV: Human immunodeficiency virus
ICER: Incremental cost effectiveness ratio
ORIC: Organizational Readiness for Implementing Change
PCAT: PMTCT cascade analysis tool
PMTCT: Prevention of mother-to-child HIV transmission
RE-AIM: Reach, Effectiveness, Adoption, Implementation and Maintenance Framework
SAIA: Systems Analysis and Improvement Approach
WHO: World Health Organization
