# Supplementary figures and images for: Scaling-up the Systems Analysis and Improvement Approach for prevention of mother-to-child HIV transmission in Mozambique (SAIA-SCALE): a stepped-wedge cluster randomized trial
Source: Implement Sci. 2019 Apr 27;14:41. doi: 10.1186/s13012-019-0889-z (PMC6487047; doi:10.1186/s13012-019-0889-z)

## Slide 1
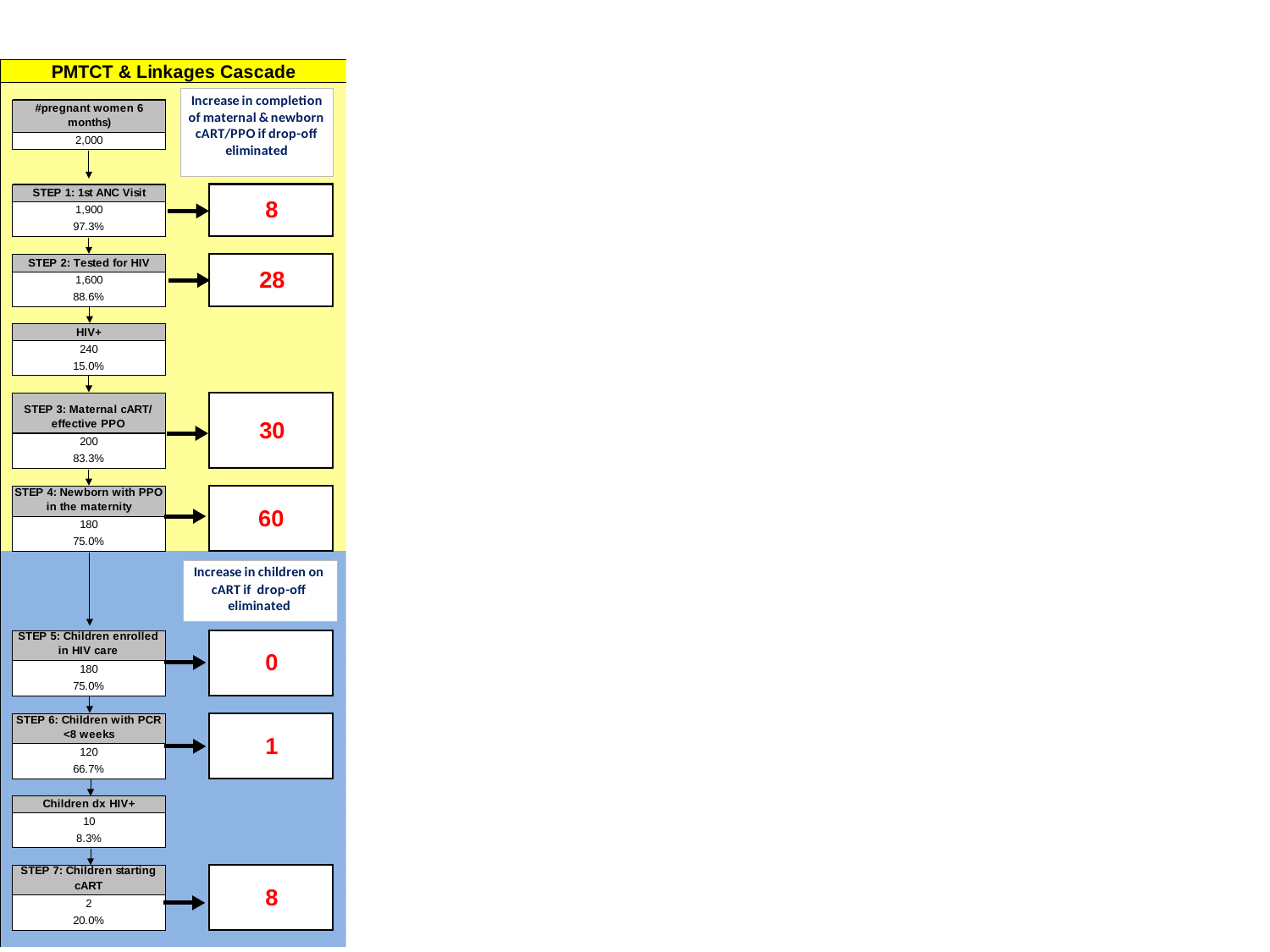

Supplement: Supplementary file 1 — Figure S1. PMTCT Cascade Analysis Tool (PCAT). Legend: Demonstrates number lost and potential gains per step (if that step improved to 100%, holding the other steps constant) for the ANC➔maternity (yellow), and postpartum (blue) cascades. ANC: Antental care; cART: Combination antiretroviral therapy; dx: Diagnosed; PPO: Prophylaxis; (PPTX 45 kb) [file 13012_2019_889_MOESM1_ESM.pptx]

## Slide 1
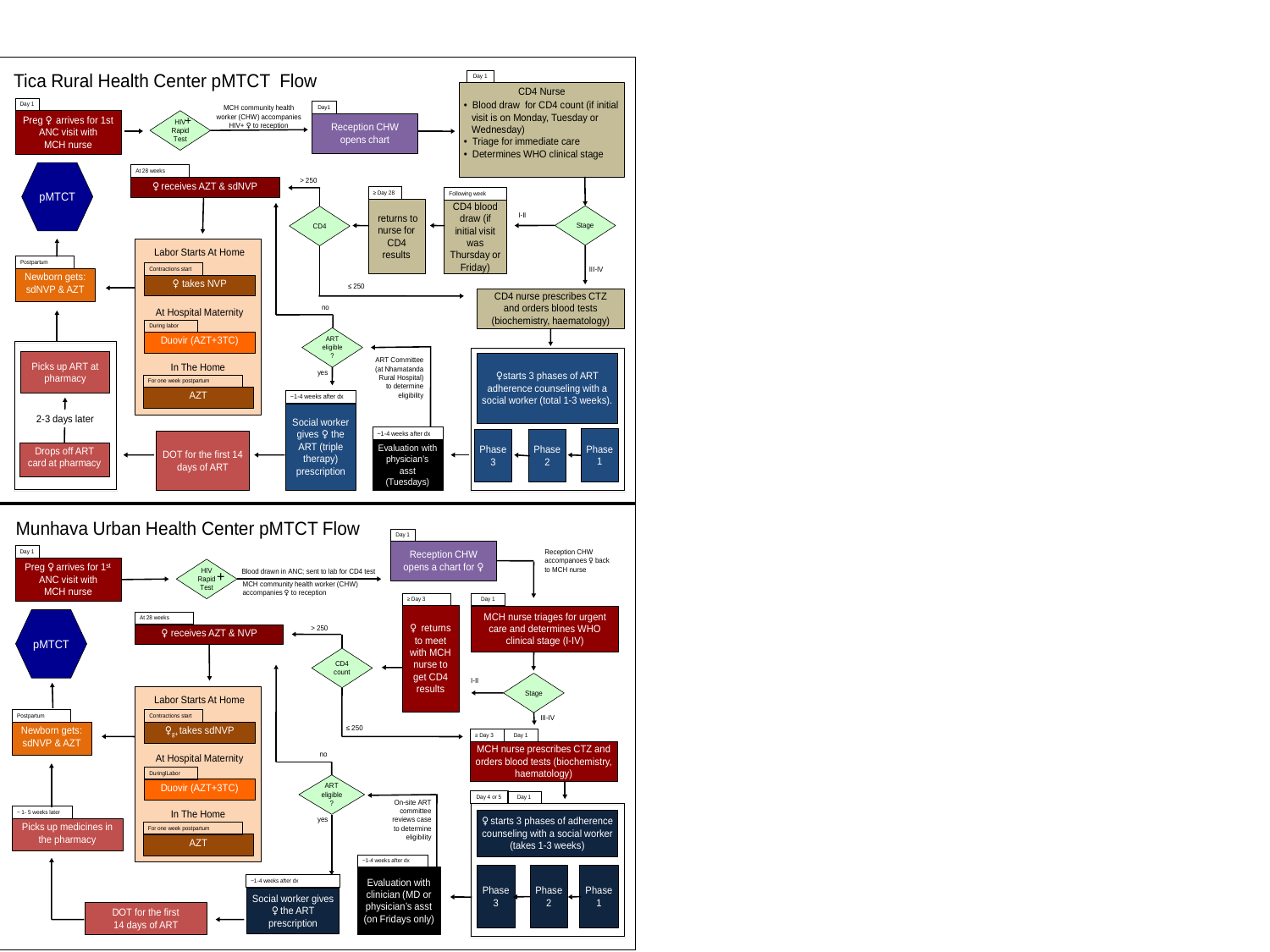

Supplement: Supplementary file 2 — Figure S2. Example of PMTCT process maps from two facilities in central Mozambique. Legend: Maps are from a medium-sized rural health center (Tica) and large urban health center (Munhava) in 2009, and demonstrate the flow of women from entry into antental care through receipt of antiretroviral prophylaxis or combination antiretroviral therapy. (PPTX 129 kb) [file 13012_2019_889_MOESM2_ESM.pptx]
